# Assessment of Mediterranean *Citrus* Peel Flavonoids and Their Antioxidant Capacity Using an Innovative UV-Vis Spectrophotometric Approach

**DOI:** 10.3390/plants12234046

**Published:** 2023-11-30

**Authors:** Rosario Mare, Roberta Pujia, Samantha Maurotti, Simona Greco, Antonio Cardamone, Anna Rita Coppoletta, Sonia Bonacci, Antonio Procopio, Arturo Pujia

**Affiliations:** 1 Department of Medical and Surgical Sciences, University “Magna Græcia” of Catanzaro, 88100 Catanzaro, Italy; mare@unicz.it (R.M.); roberta.puj@gmail.com (R.P.); simona.greco001@studenti.unicz.it (S.G.); pujia@unicz.it (A.P.); 2 Department of Clinical and Experimental Medicine, University “Magna Græcia” of Catanzaro, 88100 Catanzaro, Italy; 3 Department of Health Sciences, Institute of Research for Food Safety & Health IRC-FSH, University “Magna Græcia” of Catanzaro, 88100 Catanzaro, Italy; tony.c@outlook.it (A.C.); annarita.coppoletta1@gmail.com (A.R.C.); 4 Department of Health Sciences, University “Magna Græcia” of Catanzaro, 88100 Catanzaro, Italy; s.bonacci@unicz.it (S.B.); procopio@unicz.it (A.P.); 5 Research Center for the Prevention and Treatment of Metabolic Diseases, University “Magna Græcia” of Catanzaro, 88100 Catanzaro, Italy

**Keywords:** polyphenols, flavonoids, citrus, UV-Vis spectrophotometer, HPLC-UV, Electron Paramagnetic Spectroscopy

## Abstract

Citrus fruits exert various beneficial health effects due to the large amount of polyphenols they contain. Citrus peels, often considered food waste, contain several health-promoting polyphenols. Among these, flavonoids have long been quantified through colorimetric assays which, if not adequately applied, can lead to conflicting results. Flavonoids possess strong antioxidant properties and can decrease circulating free radicals, thereby reducing oxidative stress phenomena. Quantifying flavonoids and properly estimating their antioxidant capacity allows us to predict plausible beneficial effects of citrus fruits on human health. The aim of this research was to analyze the advantageous phenolic compounds found in the peels of citrus fruits commonly found in the Mediterranean region. The objective was to measure their antioxidant capacity and ability to neutralize free radicals. To achieve this purpose, UV-visible spectrophotometric analyses, liquid chromatography (LC) and Electron Paramagnetic Spectroscopy (EPR) were utilized and compared, finally suggesting an innovative approach for assessing the overall flavonoid content by the nitrite-aluminum assay. HPLC data demonstrated that hesperidin was the most abundant flavonoid in all peel extracts except for orange peels, in which naringin was the predominant flavonoid. The total flavonoid content was greater than 1.3 mg/mL in all extracts, with tangerine and orange yielding the best results. Citrus peel polyphenols exerted strong antioxidant and free radical scavenging effects, inhibiting up to 75% of the free radicals used as reference in the EPR analyses.

## 1. Introduction

Citrus fruits belong to the *Rutaceae* family that is widely abundant in the Mediterranean area. These fruits are known for their bright and refreshing flavors, high vitamin C content, versatile culinary uses, as well as their nutritional and health benefits. Despite being available in different seasons and widely consumed in various forms, such as fresh or processed juices, little attention has been paid to the fruits’ peels. Often considered industrial waste, the peels remain unused, despite containing various beneficial molecules [1].

Most of citrus fruits’ positive effects are related to their high content of polyphenols, the most abundant of which are flavonoids, which are now receiving considerable attention from the scientific community for their potential health benefits [2]. Indeed, they are known for their antioxidant properties [3], cardiovascular support [4], anti-inflammatory effects [5] and potential anticancer properties which also include the prevention of several chronic diseases [6].

Different analytical methods can be employed to quantify flavonoids. Despite their high accuracy, many of these methods entail high costs and lengthy analysis times, especially for chromatographic techniques such as high-pressure liquid chromatography (HPLC) [7] and mass spectrometry (MS) [8]. For all these reasons, researchers seek to prefer faster and cheaper techniques such as spectrophotometric analyses, which guarantee satisfactory precision and accuracy, as well as fast and economic analyses through direct measurements or colorimetric assays [9].

Colorimetric assays are versatile approaches applicable to different substrates and extracts with suitable modifications. However, certain modifications can result in misleading results over time, as observed in the case of the nitrite-aluminum assay widely used in the literature to quantify flavonoids. Specifically, variations in terms of reagents, wavelength or reference standard molecules may lead to inaccurate results that do not reflect the real content of flavonoids [10].

The purpose of this study was to characterize the beneficial phenolic molecules present in citrus fruit peels, which are widespread in the Mediterranean area. Additionally, the study aimed to quantify their antioxidant and free radical scavenging abilities. To achieve these goals, UV-visible spectrophotometry, liquid chromatography (LC) and Electron Paramagnetic Spectroscopy (EPR) were employed and compared. The study ultimately suggests an innovative approach to quantify the total flavonoid content (TFC) through the nitrite-aluminum colorimetric assay.

## 2. Results

### 2.1. Analyses of Standard Flavonoid Molecules

**Figure 1:**
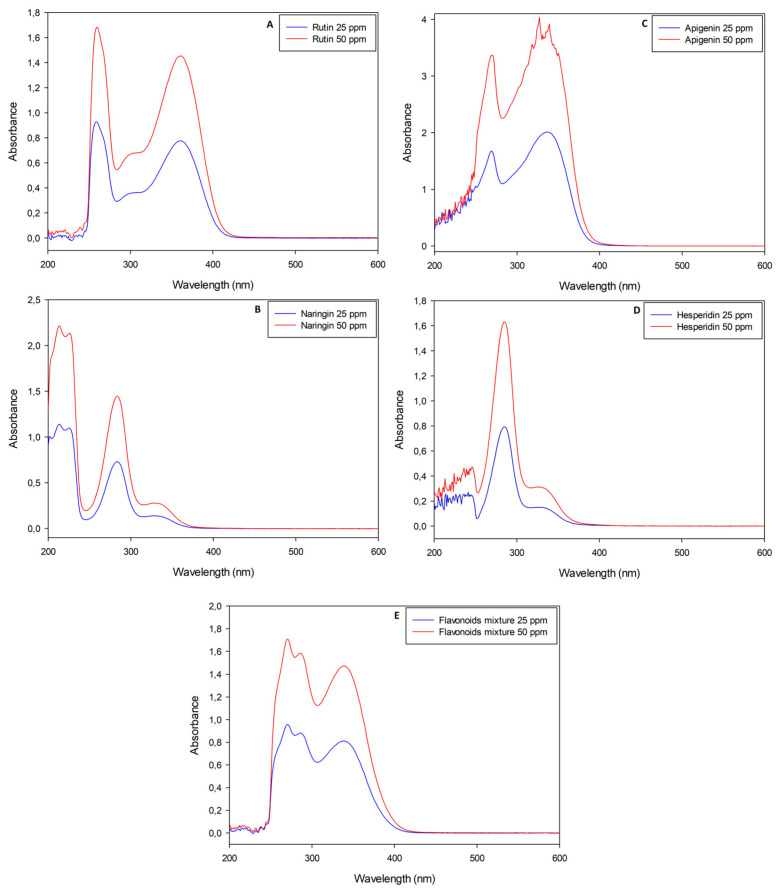
UV-Visible spectrophotometric analyses of pure flavonoid standard solutions (rutin, naringin, apigenin and hesperidin, respectively, for panels (**A**–**D**)) with wavelengths between 200 nm and 600 nm. Panel (**E**) is the analysis of a mixture obtained with all standard molecules previously listed in weight ratio 1:1:1:1.

Naringin, rutin, apigenin and hesperidin pure standard solutions were analyzed with the UV-Vis spectrophotometer before and after performing the nitrite-aluminum colorimetric assay. Standard solutions were diluted in order to obtain maximum absorbance values in agreement with the Lambert–Beer law, although apigenin had an absorbance approximately double that of the other molecules (Figure 1C).

**Figure 2:**
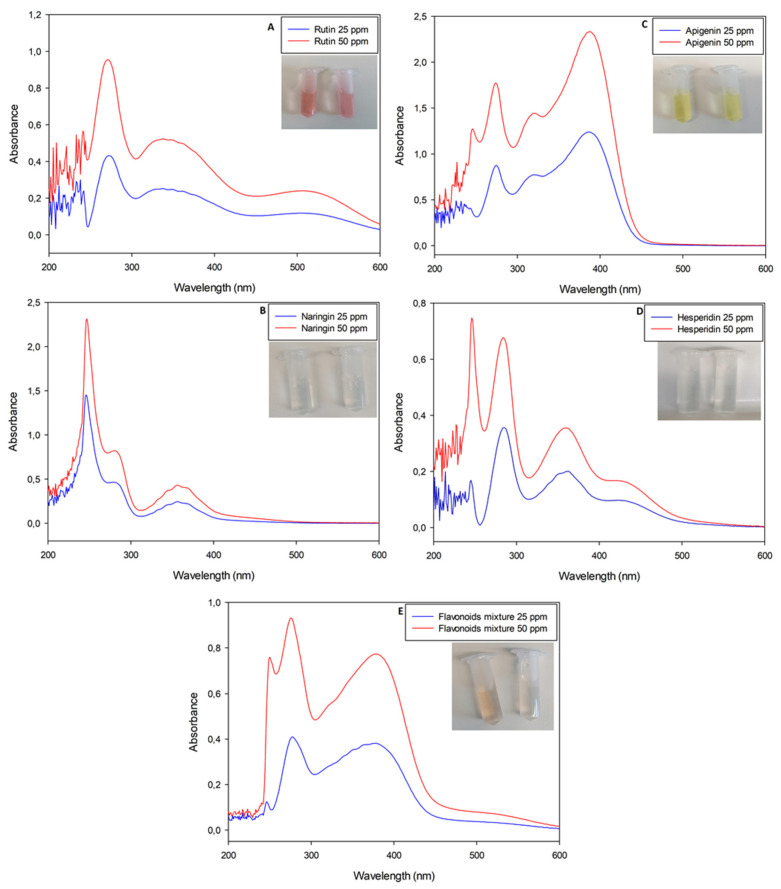
UV-Visible spectrophotometric analyses of nitrite-aluminum assay executed with pure flavonoid standard solutions (rutin, naringin, apigenin and hesperidin, respectively, for panels (**A**–**D**)). Panel (**E**) is the analysis of a mixture obtained with all standard molecules previously listed in weight ratio 1:1:1:1.

Pure flavonoid solutions showed peculiar and concentration-dependent profiles, with two or three main peaks between the near-UV and visible regions (λ < 400 nm). In detail, rutin stock solutions had a maximum absorbance (Abs_MAX_) value at λ~360 nm (Figure 1A); apigenin stock solutions had their Abs_MAX_ value at λ~340 nm (Figure 1C); naringin and hesperidin stock solutions showed similar spectra with Abs_MAX_ values at λ~330 nm (Figure 1B,D). A mixture of all the standard molecules was analyzed with the aim of simulating the simultaneous presence of different flavonoids in natural extracts. The resulting sample showed an almost overlapping spectrophotometric profile with an Abs_MAX_ value at λ~340 nm (Figure 1E). The nitrite-aluminum assay was applied to all solutions listed above and led to slight variations in terms of Abs_MAX_ wavelengths, as depicted in Figure 2.

Interestingly, all solutions presented a slight yellow color, except for rutin solutions which shifted to a reddish color as a function of the molecule concentration (Figure 2).

In particular, the nitrite-aluminum assay of the rutin solution was the only sample with a characteristic red–purple color variation, justifying its plausible quantification at λ = 510 nm. Apigenin solution shifted to a dark yellow, while naringin and hesperidin retained a light yellow color. The flavonoid mixture did not move to a red color, with the spectrophotometric profile not showing a characteristic peak at λ = 510 nm.

**Figure 3:**
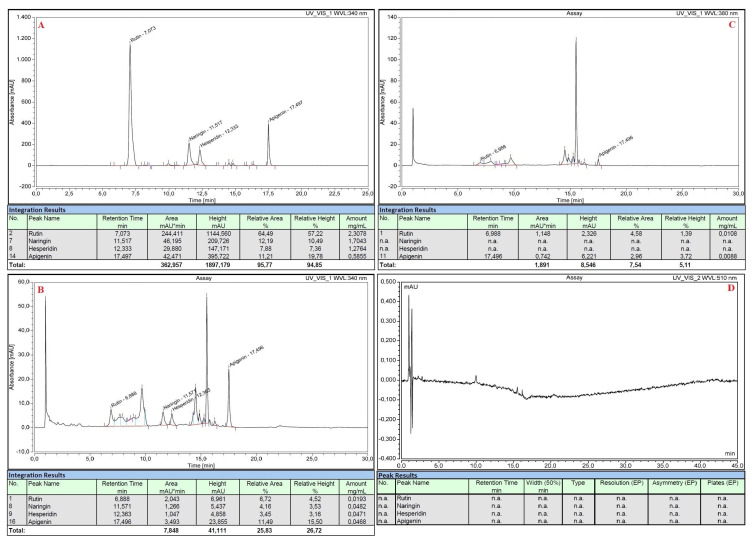
HPLC analyses of standard pure flavonoid mixture before (panel (**A**)) and after (panel (**B**–**D**)) executing the nitrite-aluminum assay with detector set at different wavelengths.

All the standard flavonoid solutions were also analyzed with HPLC before and after the execution of the nitrite-aluminum colorimetric assay. Rutin, naringin, hesperidin and apigenin were efficiently separated by the chromatographic method with a standard error of 4% and showed retention times of 7.073 min, 11.517 min, 12.333 min and 17.497 min, respectively (Figure 3A). The nitrite-aluminum assay resulted in the correct reduction or disappearance of the previously obtained signals, both at λ = 340 nm (Figure 3B) and at λ = 380 nm (Figure 3C), and the simultaneous onset of new characteristic peaks at retention times of ~9.5, ~14.5 and ~15.5 min, respectively (Figure 3B,C). No signals were detected at λ = 510 nm in the HPLC analyses (Figure 3D).

### 2.2. Citrus Peel Extracts Characterisation

**Figure 4:**
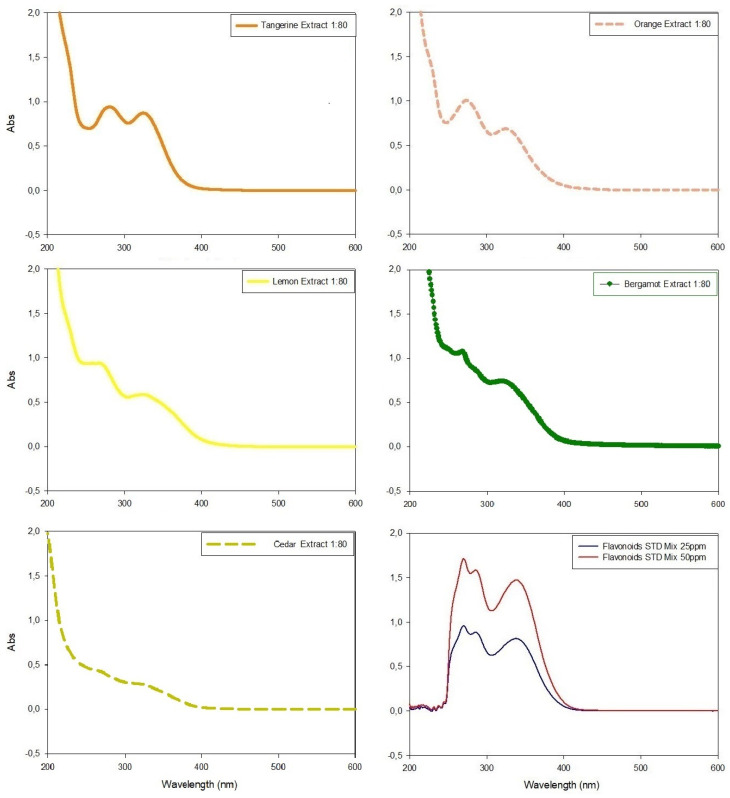
UV-Visible spectrophotometric analyses of citrus extracts in wavelengths between 200 nm and 600 nm and their comparison with the spectrophotometric data obtained with standard flavonoid mixture. All extracts were diluted (molar ratio 1:80) with extraction medium before analyses in order to respect Lambert–Beer law.

**Figure 5:**
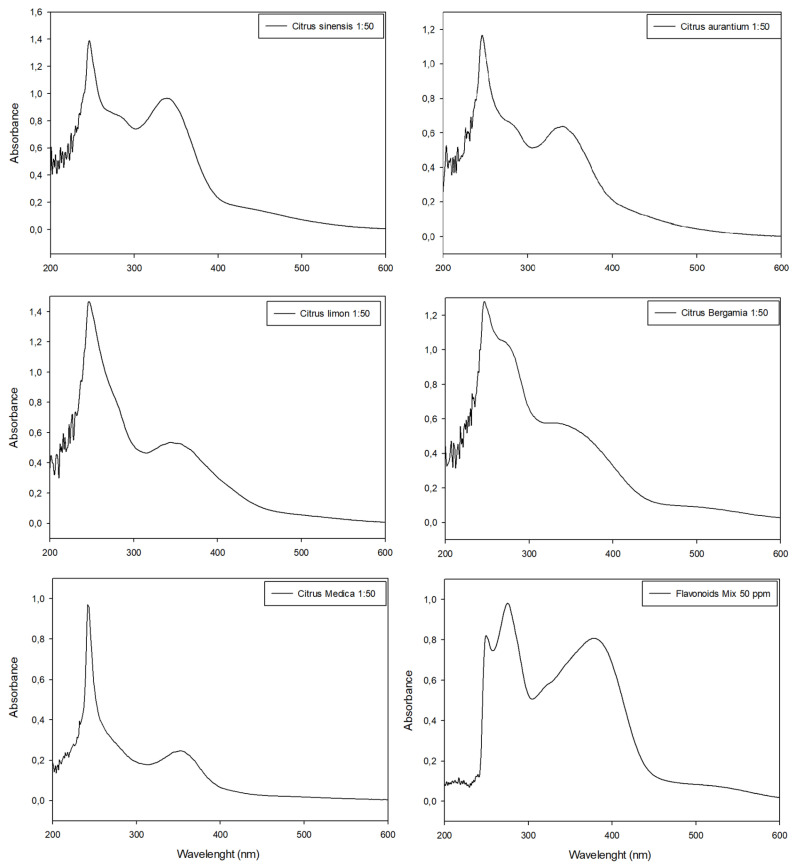
UV-Visible spectrophotometric analyses of citrus peel extracts analyzed with nitrite aluminum assay for flavonoids quantification. Last panel shows the data obtained with standard flavonoid mixture used for comparison. All extracts were diluted (molar ratio 1:50) with extraction medium before analyses in order to respect Lambert–Beer law.

Citrus peel extracts were analyzed with HPLC (Supplementary Materials) and the UV-Vis spectrophotometer in scansion mode (200 nm < λ < 700 nm) before (Figure 4) and after (Figure 5) the colorimetric assay application.

Despite the high cost and longer analysis times, HPLC tests were mandatory for confirming the data obtained with the UV-vis spectrophotometer and providing more detailed and analytically correct information about each individual sample.

HPLC analyses confirmed that rutin, naringin, hesperidin and apigenin used as reference standards were detectable in all citrus peel extracts, albeit in different proportions (Supplementary Material). Even considering the only standard molecules available as references, bergamot showed a concentration of flavonoids up to 0.8915 ± 0.0081 mg/mL and, in any case, represented the extract with the highest number of signals identified (28 peaks). Orange, lemon and tangerine had flavonoid concentrations up to 0.5130 ± 0.0237, 0.5198 ± 0.0007 and 0.4575 ± 0.0086 mg/mL, respectively, although the numbers of detectable signals were 16, 27 and 24 peaks, respectively. Also, in this case, the analyses conducted on the cedar extract provided the worst results, revealing a maximum flavonoid concentration of only 0.1395 ± 0.0021 mg/mL and with 23 peaks identified. Hesperidin was the most abundant flavonoid in all peel extracts except for orange peels, in which naringin was predominant (Table 1 and Supplementary Material).

In all spectrophotometric analyses of pure extracts, two peaks were clearly distinguishable, the highest between 270 nm and 280 nm and the minor peak at λ~325 nm, except for tangerine extracts which showed similar Abs intensity in both peaks (Figure 4). The comparison of absorbance values at ~330 nm evidenced tangerine to be the extract with the highest concentration of phenolic compounds (Abs = 0.872), followed by bergamot, orange and lemon extracts (Abs = 0.721, Abs = 0.688 and Abs = 0.586, respectively), while cedar extract had the lowest value (Abs = 0.271) (Figure 4).

The TPC assay confirmed data obtained at λ~325 nm with tangerine peels showing the highest amount of phenolic compounds compared to other citrus peel extracts, with a chemical structure similar to gallic acid (0.375 ± 0.012 mg GAE/mL). Bergamot and orange extracts showed 0.350 ± 0.016 and 0.338 ± 0.037 mg GAE/mL, respectively, while lemon and cedar extracts contained 0.305 ± 0.038 and 0.164 ± 0.019 mg GAE/mL, respectively (Table 2).

The nitrite-aluminum colorimetric assay was used to discriminate and quantify total flavonoids in citrus peel extracts. All samples provided positive results, thus confirming the ability of this assay to detect flavonoids. However, none of the citrus peel extracts showed color changes to red or showed the presence of a characteristic peak at 510 nm. Conversely, a characteristic peak of flavonoids was detected in 340 nm < λ < 370 nm in all extracts as well as in standard molecule solutions (Figure 5).

The flavonoid colorimetric assay confirmed the TPC trend. In fact, the tangerine peel extract had the highest flavonoid content (Figure 5; Abs = 0.9634 at λ~340nm); orange, bergamot and lemon peel extracts showed an intermediate flavonoid content (Figure 5; 0.55 < Abs < 0.64 at λ~340nm) while the cedar peel extract evidenced the lowest amount of flavonoids in comparison with other extracts (Figure 5; Abs < 0.5 at λ~340 nm). These results were not confirmed using the traditional quantification method. The total flavonoid content of all citrus extracts according to the nitrite-aluminum assay is listed in Table 3, along with the comparison between the proposed approach and the traditional method of quantification.

All samples derived from the application of the nitrite-aluminum assay were also analyzed with HPLC, using the same analytical method but with an increased run time, in order to identify other plausible molecules formed in the colorimetric assay. In any case, the oxidation of nitrite salts and the conjugation with aluminum cations led to a decrease in or disappearance of peaks previously detected. Interestingly, the colorimetric assay also produced new signals detectable at 340 nm with different absorbance intensities and a retention time between 20 and 25 min in all extracts investigated (Supplementary Material).

### 2.3. Antioxidant Activity of Citrus Peel Extracts

The antioxidant activity of all citrus peel extracts was estimated using a colorimetric assay detecting the capability of each extract to inactivate a free radical, in this case, DPPH. The results obtained are listed in Table 4. Interestingly, the antioxidant activity shown by the citrus peel extracts was not always directly proportional to the total amount of flavonoids previously detected. Indeed, the best antioxidant activity was detected in orange extracts (73.93 ± 3.14 mean %I), followed by lemon, tangerine and bergamot showing 71.55 ± 2.16, 54.66 ± 3.49 and 34.43 ± 0.74 mean %I, respectively. Conversely, cedar peel extracts did not exceed 28.55 ± 1.1 mean %I of free radical inhibitory activity.

### 2.4. Scavenging Activity of Citrus Peel Extracts

The scavenging activity of the extracts against DPPH radicals has been evaluated through Electronic Paramagnetic Resonance (EPR). The decrease in EPR signal intensity was related to the free radical scavenging activity of the extracts.

**Figure 6:**
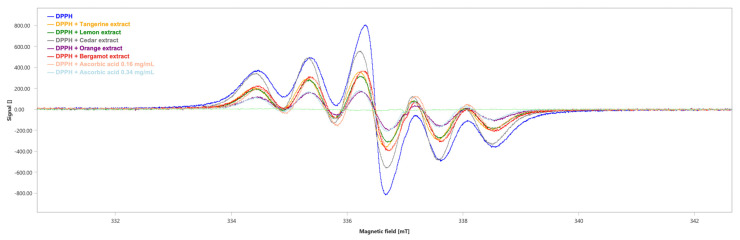
EPR spectra of DPPH in the absence (blue) and presence of the tested extracts. Ascorbic acid has been used as positive control. mT: millitesla.

We obtained the six-line pattern EPR spectrum of the DPPH (Figure 6) with an integrated spectral area (∫) value of 1754 ± 3 a.u. (Table 5).

All the tested extracts showed DPPH radical scavenging capability, although the orange extract showed the highest antioxidant capability with a scavenger percentage of 75.42% (∫ = 431 ± 3, Table 5), followed by the lemon, tangerine and bergamot extracts with a scavenger percentage of 59.18% (∫ = 716 ± 3, Table 5), 55.30% (∫ = 784 ± 3, Table 5) and 51.03% (∫ = 859 ± 3, Table 5), respectively. Lastly, cedar showed the lowest antioxidant ability with a scavenger percentage of 26.62% (∫ = 1287 ± 3, Table 5). As positive controls, the scavenger percentages of ascorbic acid were 55.19% at the concentration of 0.16 mg/mL (∫ = 786 ± 3, Table 5) and 75.65% at the concentration of 0.34 mg/mL (∫ = 427 ± 2, Table 5).

## 3. Materials and Methods

### 3.1. Materials

Powders of sodium nitrite, aluminum chloride, sodium hydroxide and 85% phosphoric acid solution as well as naringin, rutin, apigenin and hesperidin used as standard molecules for stock solutions were purchased from Merck Sigma-Aldrich (Milan, Italy). Methanol, ethanol, dimethyl sulfoxide and all other reagents were of analytical grade (Carlo Erba, Milan, Italy). Quartz cuvettes with prefixed optical paths (1 cm) were used for all spectrophotometric analyses and were purchased from Exacta Optech (Monza, Italy).

The citrus fruits oranges, lemons, clementine, cedar and bergamot (known in the scientific field as *Citrus aurantium*, *Citrus limon*, *Citrus sinensis*, *Citrus medica* and *Citrus bergamia*, respectively) were kindly and freely granted by local farmers from south Italy based on their seasonal availability.

### 3.2. UV-Vis Spectrophotometer Analyses

The spectrophotometer apparatus used was a ThermoFisher Scientific Genesys^TM^ 150 equipped with a xenon lamp, VISION lite 5 software for remote computer control, and different on-board applications, including quantitative analyses and a scanning mode of the complete spectrum. The apparatus can detect wavelengths between 190 and 1100 nm with high resolution and precision (±0.1 nm). Quartz cuvettes with a 1 cm optical path and a maximum volume of 2 mL were used in all analyses.

In detail, the scansion mode with wavelengths between 200 and 600 nm was used for pure extract characterization and flavonoids quantification, while the fixed wavelength mode was used for total phenolic content (760 nm) and antioxidant activity (518 nm) assays as better described below.

### 3.3. HPLC Analyses

In order to verify and confirm the results obtained using UV-Vis spectrophotometric analyses, the standard flavonoid solutions and citrus peel extracts were analyzed with high-performance liquid chromatography (HPLC) before and after performing the nitrite-aluminum colorimetric assay. The HPLC apparatus consisted of a ThermoFisher Scientific Vanquish System Base equipped with a quaternary pump, split sampler, thermostatic column compartment, a fluorimeter and a UV/VIS variable wavelength detector (ThermoFisher Scientific-Rosano, Milan, Italy). Chromeleon^®^ software version 7.2 was used for data processing and was purchased from ThermoFisher Scientific (Rosano, Milan, Italy). The column used was an Acclaim^®^ 120 reverse phase C18 (100–4.6 mm), with a particle size of 5 µm. The mobile phase was H_3_PO_4_ 10mM aqueous solution and ACN with ratios ranging from 85:15 to 60:40 in 20 min during each analysis. The column temperature was set at 30 °C with a forced air cooling system, and signals were acquired at 340 nm, 380 nm and 510 nm. Peak identification was conducted by comparing the retention times with those obtained with standard solutions of rutin, naringin, hesperidin and apigenin. Suitable calibration curves (min conc. 31.25 ppm and max conc. 500 ppm–r^2^ > 0.998 in all cases; Supplemental Figure S1) were used for the identification of flavonoids contained in the samples. A 5 µL aliquot of each sample was injected into the HPLC apparatus, and the total acquisition time was set to 30 min.

### 3.4. Citrus Peel Extracts (CPE)

The phenolic compounds contained in *Citrus* peels were extracted as previously described in the literature by Stalikas and coworkers with slight modifications [11]. Briefly, the fruits were manually peeled to obtain a very thin peel composed of the endocarp and glandular tissue of the fruits. The peels were incubated with the extraction medium in a ratio of 1:5 weight/volume and left to macerate for 24 h. The extraction medium used was a hydro-alcoholic solution (ethanol/milliQ^®^ water (Merk Millipore, Darmstadt, Germany)) with a volume ratio of 7:3.

After incubation, the peels were removed, and the extract solutions were purified using filtration through 0.45 µm cellulose filters before being collected. The colored and fragrant extracts of citrus were subsequently analyzed with the UV-Vis spectrophotometer both before and after being subjected to different colorimetric assays, as described below. Suitable dilution factors were properly applied in each analysis.

### 3.5. Total Phenolic Content (TPC)

The total phenolic content of all *Citrus* peel extracts was obtained as previously described in the literature [12]. Briefly, 50 µL of the sample was added to 250 µL of Folin–Ciocalteu′s phenol reagent. The resulting mixture was vortexed and incubated for 5 min at room temperature. Subsequently, 500 µL of a sodium carbonate aqueous solution was added to the sample, followed by another incubation at room temperature for 25 min [13]. During each incubation procedure, all samples were protected from light. Absorbance at λ = 760 nm was detected immediately after the incubation step. Gallic acid was used as a reference molecule for the calibration curve at concentrations between 0.1 and 0.5 mg/mL. The results are listed in Table 2 and described as milligrams equivalent of gallic acid per mL extract (mg GAE/mL–calibration curve y = 4.804x + 0.006; r^2^ = 0.996).

### 3.6. Flavonoid Extracts and Quantification

The standard flavonoid solutions and citrus extracts were analyzed using a UV-Vis spectrophotometer following the colorimetric assay described by Lenucci in 2006 [14]. Briefly, stock solutions of naringin, apigenin, hesperidin and rutin were prepared by dissolving powders in suitable solvents, following the recommendations of the supplier. Stock solutions were subsequently diluted in order to obtain fixed concentrations, and 1 mL of the sample was incubated with 60 µL of sodium nitrite aqueous solution (5% *w*/*v*) and allowed to react for 5 min. Then, 120 µL aluminum chloride aqueous solution (10% *w*/*v*) was added, and after 5 min of incubation, 0.40 mL of sodium hydroxide (1 M) and 0.42 mL of distilled water were added to the mixture to neutralize the pH. The final volume in each sample was 2 mL.

The samples were immediately measured with a spectrophotometer UV-Vis (Thermo Scientific-Genesys^®^ 150, Milan, Italy) set to scan mode to quantify absorbance across all wavelengths (λ) between 200 and 600 nm. Specific peaks and wavelengths were selected as a function of the flavonoids investigated.

Suitable calibration curves were calculated with absorbance values obtained using rutin or a mixture of naringin, rutin, apigenin and hesperidin in the weight ratio 1:1:1:1 (Supplemental Figure S2–C_MIN_ = 6 ppm; C_MAX_ = 75 ppm; λ~510 nm; y = 0.005x + 0.0012; r^2^ = 0.994 and λ~375 nm; y = 0.0153x + 0.0615; r^2^ = 0.994, respectively).

### 3.7. Antioxidants of Citrus Peel Extracts

The antioxidant activity of all citrus extracts was calculated using a colorimetric assay based on the capability of inhibiting the free radical 2,2-Diphenyl-1-picrylhydrazyl (DPPH) as previously described in the literature [15].

Briefly, 50 µL extract and 5 mL of 0.004% (*w*/*v*) methanol solution of DPPH were mixed, vortexed and incubated in the dark for 30 min at room temperature. Thereafter, the absorbance of each mixture was read with a UV/VIS spectrophotometer at 518 nm. The blank was 80% (*v*/*v*) methanol, and a DPPH solution was used as the negative control while an L-ascorbic acid solution (5 mg/mL) was used as the positive control. The percentage of DPPH inhibition was calculated using the following formula:

I(%) = [(A_0_ − A_1_)/A_0_] × 100 (1)

A_0_ is the absorbance of the negative control and A_1_ is the absorbance of the extracts/standards.

### 3.8. Scavenging Activity of Citrus Peel Extracts

The scavenging activity of the various extracts against DPPH radicals was also evaluated through Electron Paramagnetic Spectroscopy (EPR) as previously described by Musolino and coworkers [16].

The capability of the extracts to neutralize DPPH radicals was assessed by adding 50 μL of the tested extract to 200 μL of 1 mM DPPH (methanolic solution). Ascorbic acid, known for its antioxidant properties, was used as the positive control at two different concentrations (0.16 mg/mL and 0.34 mg/mL).

After 1 min of reaction, the EPR spectra were acquired using a Bruker Magnettech ESR5000 (Bruker Biospin MRI GmbH, Ettlingen, Germany) set with the following parameters: 9.43 GHz X band, 0.05 mT modulation amplitude, 336.64 mT central field, 12.00 mT sweep, 30 s sweep time, 100 Khz modulation frequency, 8 accumulations and 20 mW microware power.

To assess the total amount of free radicals in each acquisition and evaluate the radical scavenging activity of the tested extracts, the spectral areas were calculated using the ESRStudio software (v. 1.74.6, Bruker Biospin, Ettlingen, Germany). The percentages of scavenging were quantified using the formula below:

scavenger % = (Ar_0_ − Ar_extract_/Ar_0_) × 100 (2)

Ar_0_ is the area of the negative control and Ar_extract_ is the area of the extracts/standards.

### 3.9. Statistical Analyses

All experiments were conducted in triplicate. Data are presented as means of three independent experiments ± standard deviation, when applicable. Data concerning all physicochemical properties of the extracts were processed using Microsoft^®^ Excel^TM^ (Office Pack 2016-Microsoft^®^ Corporation, Redmond, WA, USA). Graphs and statistical tests for UV/VIS spectrophotometric analyses were obtained using SigmaPlot 12.0 (Systat Software Inc., 2011, Chicago, IL, USA).

## 4. Discussion

Polyphenols are important molecules contained in vegetables and fruits which abound in citrus fruits and are directly linked to health benefits. Therefore, it is crucial to quantify them correctly. Among the different polyphenol extraction methods described in the literature, the hydroalcoholic solutions we used represent the most widespread, rapid and economically advantageous [17].

Flavonoids are polyphenols that have been quantified for decades through the nitrite-aluminum colorimetric assay. However, the many changes made to the original protocol over time can lead to misleading and inaccurate results [10]. In fact, several authors in the literature replace sodium nitrite with nitrate salts which do not have the same oxidizing effect. Additionally, they use rutin or quercetin as a single reference molecule and consider fixed wavelengths in a range between 480 and 510 nm [10]. This approach, as underlined by Sisa and his collaborators, would also exclude different flavonoids from being quantified because of their aromatic rings which contain carbonyl chromophores absorbing at λ~350 nm [18].

In agreement with the scientific literature, our results demonstrate that all flavonoids, both individually and in mixtures, possess a pale yellow color and a similar spectrophotometric profile before performing the colorimetric assay (Figure 1). However, after incubation with nitrite and aluminum, the rutin solution (Figure 2A) was the only sample undergoing a color change, a phenomenon that did not occur in all the other samples due to the lack of the catechin ring. Interestingly, the mixture of flavonoids also did not undergo color variations, just as there was no appearance of any characteristic peak at 510 nm, despite the presence of rutin in the analyzed solution (Figure 2E). The observed phenomenon can be traced back to the simultaneous presence of molecules such as apigenin, which, having a greater signal intensity, can alter or even mask peaks and signals of the other molecules [19].

This involves a variation in the spectrum that would not be appreciable if the sample had been analyzed at a single wavelength, but it becomes evident by scanning the entire visible spectrum. In detail, before performing the nitrite aluminum assay, standard flavonoid pure solutions had maximum absorbance values at λ~340 nm, even when mixed (Figure 1A, Figure 2A and Figure 3A). The nitrite-aluminum assay led to a shift in this signal due to the nitrite-mediated oxidation of carbonyl groups (Figure 2). Indeed, the HPLC analyses also demonstrated a better intensity at λ~380 nm (Figure 3C) than that detected at λ~340 nm (Figure 3B), while no signal was detected at 510 nm.

Citrus peels possess potent antioxidant properties due to their rich content of flavonoids and other polyphenols known for scavenging free radicals and reducing oxidative stress [20]. All citrus peel extracts (CPE) presented a spectrophotometric profile with a trend similar to the standard flavonoid mixture. Interestingly, the total phenolic content of the cedar peel extract was almost half compared to other species regardless of the technique used, probably due to the different structure of its peel, characterized by a much thicker and more fibrous mesocarp than other citrus fruits. Moreover, the TPC and TFC assays showed a similar trend, with tangerine peels (*Citrus sinensis*) always providing the best results and alternately followed by bergamot (*Citrus bergamia*) and orange (*Citrus aurantium*). In fact, the extracts obtained from the peel have been shown to contain a high quantity of phenols with a structure similar to gallic acid (TPC results up to 0.375 ± 0.012 mg/mL) as well as flavonoids (TFC results up to 2.946 ± 0.003 mg/mL).

In agreement with the literature data, citrus peel extracts have shown strong radical neutralization and radical elimination capabilities, thus proposing themselves as plausible valid supplements and nutraceuticals capable of contributing to promoting human health [21]. In fact, citrus peels have been shown to enhance enzymatic and non-enzymatic antioxidant defenses, offering potential therapeutic applications against oxidative-related diseases [22]. Moreover, different studies underscore the potential use of citrus peel extracts in functional foods and nutraceuticals, highlighting their role in combating oxidative damage [23]. HPLC analyses suggest the antioxidant and scavenging effects should be related to hesperidin and naringin, which were the most abundant flavonoids in citrus peel extracts. This could also be the rationale why orange peel extracts showed the best antioxidant and free radical scavenger power (>70% in both cases) in comparison with all other extracts.

The results gathered suggest that Mediterranean citrus peels are a source of functional molecules enabling the classification of citrus fruits based on the beneficial compounds they contain. Until now, the available information in the literature has been limited to descriptive reviews where authors compile and integrate results from various studies, but nobody characterized or compared citrus species or individual plant organs and tissues [24,25].

## 5. Conclusions

Spectrophotometric analyses and colorimetric assays represent valid approaches for analyzing and cataloging herbal and plant extracts.

As in the past, the total flavonoid content can be quantified via nitrite-aluminum assay, but the analysis of the complete visible spectrum and the use of a molecules mixture as the reference standard represent a preferable approach for this purpose if compared to the traditional method.

HPLC allows quantifying single flavonoids, but it requires many standard molecules, high costs and long analyses or requires to be conjugated to a mass spectrometer (MS).

Our UV-Vis spectrophotometric analyses could represent a valid alternative for quantifying total flavonoid content as it is fast and cost-effective.

Mediterranean citrus peels, often considered industrial waste, contain multiple polyphenols that are beneficial for health and have high antioxidant power. The use of citrus peel extracts as nutraceuticals or food supplements represents a valid aid in contrasting pathologies characterized by inflammatory processes due to oxidative stress and the production of free radicals.

Future studies should aim to validate our findings and compare them to the flavonoid content within dried citrus peel extracts.

## 6. Limits of the Study

Despite the promising results achieved in this study, it has some limitations that should be addressed in future research.

In fact, the availability of mass spectroscopy data using a greater number of reference standards should allow obtaining a more complete flavonoid profile. Although all the citrus fruits were taken and analyzed in the period of maximum ripeness, the results achieved are susceptible to natural variability due to the ripening stages and the balsamic periods of the individual plant species, parameters that would be interesting to study and evaluate in subsequent projects. Moreover, the study of antioxidant activity using different techniques could provide deeper information about the ROS scavenging power of citrus peels.

## Figures and Tables

**Table 1:** The best HPLC quantification of flavonoids in citrus peel extracts.

	Rutin RT = 6.97 min	Naringin RT = 11.217 min	Hesperidin RT = 12.324 min	Apigenin RT = 19.08 min	Total
	mg/mL ± SD	mg/mL ± SD	mg/mL ± SD	mg/mL ± SD	mg/mL ± SD
Lemon extract	0.0379 ± 0.0012	0.2183 ± 0.0019	0.2273 ± 0.0017	0.0362 ± 0.0017	0.5198 ± 0.0007
Orange extract	0.0571 ± 0.0007	0.2641 ± 0.0141	0.1787 ± 0.0050	0.0130 ± 0.0039	0.5130 ± 0.0237
Tangerine extract	0.0044 ± 0.0009	0.1235 ± 0.0008	0.3214 ± 0.0064	0.0081 ± 0.0004	0.4575 ± 0.0086
Cedar extract	0.0142 ± 0.0005	0.0344 ± 0.0007	0.0578 ± 0.0015	0.0329 ± 0.0006	0.1395 ± 0.0021
Bergamot extract	0.0120 ± 0.0017	0.1651 ± 0.0011	0.6881 ± 0.0078	0.0262 ± 0.0008	0.8915 ± 0.0081

RT = retention time; SD = standard deviation.

**Table 2:** Total phenolic content of citrus hydro-alcoholic extracts.

Samples	Mean Abs_760 nm_ ± SD	Mean GAE (mg/mL) ± SD
Tangerine	1.808 ± 0.058	0.375 ± 0.012
Orange	1.629 ± 0.177	0.338 ± 0.037
Lemon	1.472 ± 0.183	0.305 ± 0.038
Cedar	0.796 ± 0.091	0.164 ± 0.019
Bergamot	1.687 ± 0.075	0.350 ± 0.016

Abs = absorbance; GAE = gallic acid equivalent; SD = standard deviation.

**Table 3:** Quantification of flavonoids in citrus extracts according to the traditional nitrite-aluminum assay (λ~510 nm) and comparison with the new approach (λ~375 nm). Dilution Factor (DF) was 1:50 in all samples.

Sample	New Approach-λ~340 nm		Traditional Assay-λ~510 nm	
	Abs_340nm_ ± SD	TFC-mg/mL ± SD	Abs_510nm_ ± SD	TFC–mg/mL ± SD
Lemon extract	0.528 ± 0.001	1.524 ± 0.005	0.054 ± 0.007	0.530 ± 0.068
Orange extract	0.635 ± 0.001	1.874 ± 0.004	0.036 ± 0.003	0.349 ± 0.031
Tangerine extract	0.963 ± 0.001	2.946 ± 0.003	0.053 ± 0.011	0.517 ± 0.101
Cedar extract	0.460 ± 0.002	1.302 ± 0.005	0.030 ± 0.003	0.286 ± 0.026
Bergamot extract	0.597 ± 0.002	1.735 ± 0.005	0.091 ± 0.008	0.902 ± 0.081

Abs = absorbance; TFC = total flavonoid content; SD = standard deviation.

**Table 4:** Antioxidant activity of citrus peel extracts.

Sample	Mean Abs_518 nm_ ± SD	Mean I(%) ± SD
Negative CRT	2.482	
Control (+) L-ascorbic Acid 5 mg/mL	0.193	
Tangerine	1.12 ± 0.09	54.66 ± 3.49
Orange	0.65 ± 0.08	73.93 ± 3.14
Lemon	0.71 ± 0.05	71.55 ± 2.16
Cedar	1.77 ± 0.03	28.55 ± 1.1
Bergamot	1.63 ± 0.02	34.43 ± 0.74

SD = standard deviation

**Table 5:** EPR spectroscopy for DPPH radical scavenging activity.

Sample	Spectral Area (∫, a.u. ± SD)	% Scavenging
DPPH	1754 ± 3	
DPPH + tangerine extract	784 ± 3	55.30%
DPPH + orange extract	431 ± 3	75.42%
DPPH + lemon extract	716 ± 3	59.18%
DPPH + cedar extract	1287 ± 3	26.62%
DPPH + bergamot extract	859 ± 3	51.03%
DPPH + Ascorbic acid 0.16 mg/mL	786 ± 3	55.19%
DPPH + Ascorbic acid 0.34 mg/mL	427 ± 2	75.65%

a.u. (arbitrary units), SD (standard deviation).

## Data Availability

The data supporting the results of this manuscript are available from the authors upon reasonable request.

## Supplementary Materials

The following supporting information can be downloaded at: https://www.mdpi.com/article/10.3390/plants12234046/s1, Figure S1. HPLC calibration curves obtained with standard solutions of rutin, naringin, hesperidin and apigenin. Minimum concentration 31.25 ppm and maximum concentration 500 ppm–r^2^ > 0.998 in all cases. Figure S2. Spectrophotometric calibration curve calculated with absorbance values obtained using rutin standard solution (λ~510 nm) or a mixture of naringin, rutin, apigenin and hesperidin in weight ratio 1:1:1:1 (λ~375 nm). Minimum concentration 6 µg/mL and maximum concentration 75 µg/mL–r^2^ > 0.99 in both cases.
