# Supplementary material for: Assessment of Mediterranean Citrus Peel Flavonoids and Their Antioxidant Capacity Using an Innovative UV-Vis Spectrophotometric Approach
Source: Plants (Basel). 2023 Nov 30;12(23):4046. doi: 10.3390/plants12234046 (PMC10708307; doi:10.3390/plants12234046)
Supplement: Supplementary file 1 [file plants-12-04046-s001.zip › plants-2700800-supplementary.pdf]

Supplementary Figures

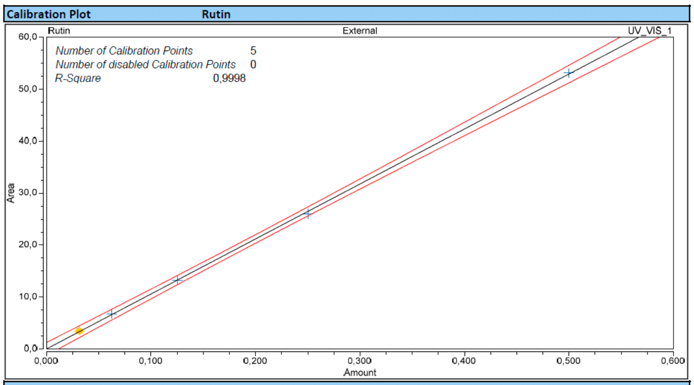

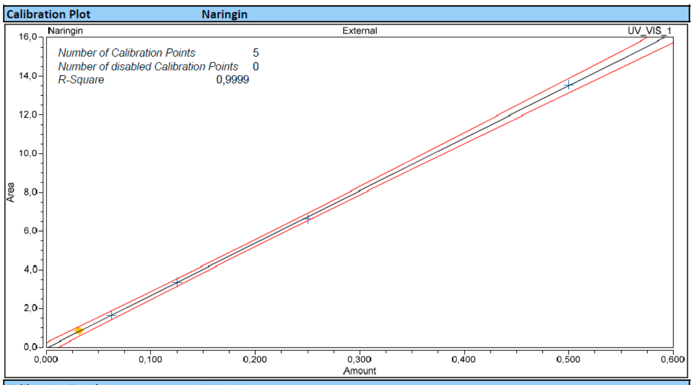

Calibration Results — Rutin

No.	Injection Name	Calibration Level	X Value UV_VIS_1 Rutin	Y Value UV_VIS_1 Rutin	Y Value UV_VIS_1 Rutin	Area mAU*min UV_VIS_1 Rutin	Height mAU UV_VIS_1 Rutin
3	S1 - 0.03125 mg/mL	1	0,0313	3,4507	3,4507	3,451	18,211
4	S2 - 0.0625 mg/mL	2	0,0625	6,7516	6,7516	6,752	35,655
5	S3 - 0.125 mg/mL	3	0,1250	13,2269	13,2269	13,227	69,457
6	S4 - 0.25 mg/mL	4	0,2500	26,0121	26,0121	26,012	136,900
7	S5 - 0.5 mg/mL	5	0,5000	53,1673	53,1673	53,167	278,948

Calibration Results — Naringin

No.	Injection Name	Calibration Level	X Value UV_VIS_1 Naringin	Y Value UV_VIS_1 Naringin	Y Value UV_VIS_1 Naringin	Area mAU*min UV_VIS_1 Naringin	Height mAU UV_VIS_1 Naringin
3	S1 - 0.03125 mg/mL	1	0,0313	0,8420	0,8420	0,842	4,085
4	S2 - 0.0625 mg/mL	2	0,0625	1,6499	1,6499	1,650	7,819
5	S3 - 0.125 mg/mL	3	0,1250	3,3730	3,3730	3,373	15,678
6	S4 - 0.25 mg/mL	4	0,2500	6,6351	6,6351	6,635	30,753
7	S5 - 0.5 mg/mL	5	0,5000	13,5653	13,5653	13,565	61,931

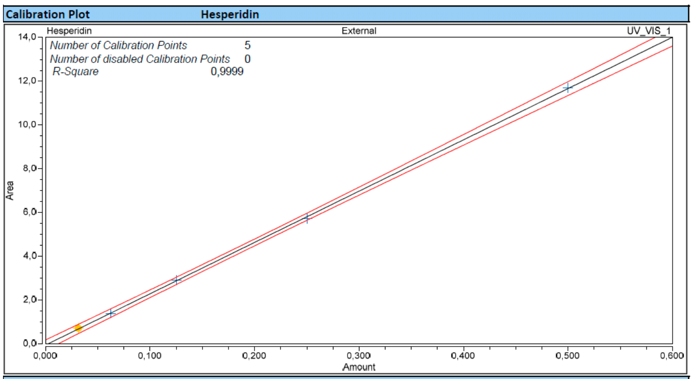

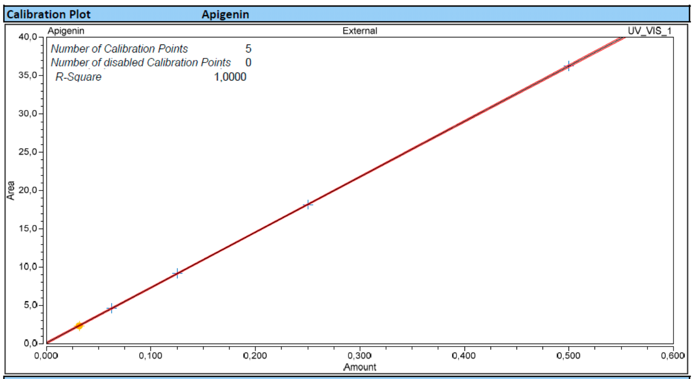

Calibration Results — Hesperidin

No.	Injection Name	Calibration Level	X Value UV_VIS_1 Hesperidin	Y Value UV_VIS_1 Hesperidin	Y Value UV_VIS_1 Hesperidin	Area mAU*min UV_VIS_1 Hesperidin	Height mAU UV_VIS_1 Hesperidin
3	S1 - 0.03125 mg/mL	1	0,0313	0,7098	0,7098	0,710	3,694
4	S2 - 0.0625 mg/mL	2	0,0625	1,3858	1,3858	1,386	7,208
5	S3 - 0.125 mg/mL	3	0,1250	2,9091	2,9091	2,909	14,416
6	S4 - 0.25 mg/mL	4	0,2500	5,7247	5,7247	5,725	28,597
7	S5 - 0.5 mg/mL	5	0,5000	11,7010	11,7010	11,701	57,766

Calibration Results — Apigenin

No.	Injection Name	Calibration Level	X Value UV_VIS_1 Apigenin	Y Value UV_VIS_1 Apigenin	Y Value UV_VIS_1 Apigenin	Area mAU*min UV_VIS_1 Apigenin	Height mAU UV_VIS_1 Apigenin
3	S1 - 0.03125 mg/mL	1	0,0313	2,3387	2,3387	2,339	21,763
4	S2 - 0.0625 mg/mL	2	0,0625	4,6435	4,6435	4,644	44,423
5	S3 - 0.125 mg/mL	3	0,1250	9,1863	9,1863	9,186	90,073
6	S4 - 0.25 mg/mL	4	0,2500	18,1722	18,1722	18,172	182,631
7	S5 - 0.5 mg/mL	5	0,5000	36,2856	36,2856	36,286	377,569

Supplemental Figure S1. HPLC calibration curves obtained with standard solutions of rutin, naringin, hesperidin and apigenin. Minimum concentration 31,25 ppm and maximum concentration 500 ppm – r2>0.998 in all cases.

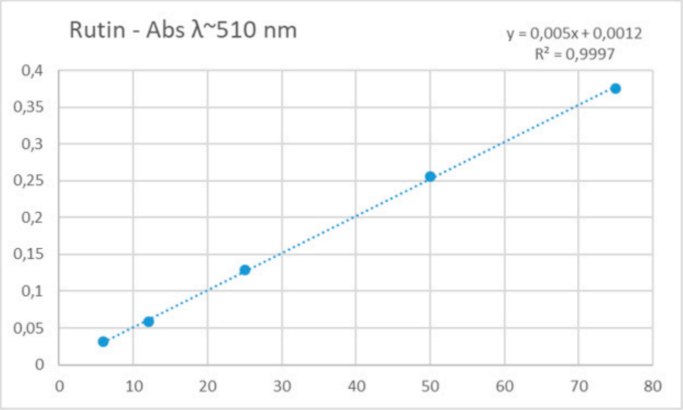

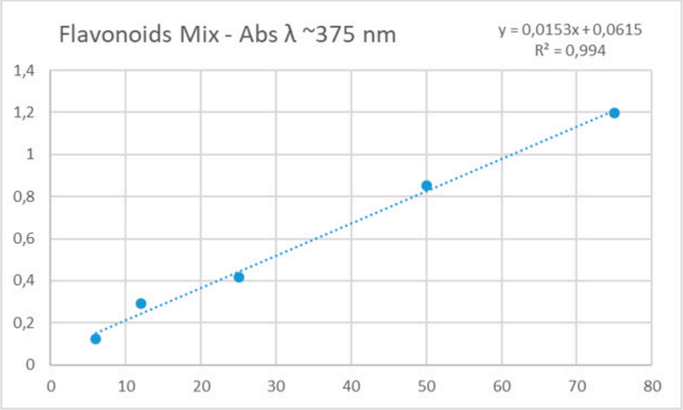

Supplemental Figure S2. Spectrophotometric calibration curve calculated with absorbance values obtained using rutin standard solution (λ~510 nm) or a mixture of naringin, rutin, apigenin and hesperidin in weight ratio 1:1:1:1 (λ~375 nm). Minimum concentration 6 μg/mL and maximum concentration 75 μg/mL – $r^2 > 0.99$ in both cases.
